# Supplementary material for: The Systems Biology Research Tool: evolvable open-source software
Source: BMC Syst Biol. 2008 Jun 29;2:55. doi: 10.1186/1752-0509-2-55 (PMC2446383; doi:10.1186/1752-0509-2-55)
Supplement: Additional file 1 — SBRT Archive. An archive of the current version of the Systems Biology Research Tool. [file 1752-0509-2-55-S1.zip › sbrt-1.4.0/doc/developers_guide/api/sbrt/kernel/fba/comp/class-use/ExtremeCurrentComputer.html]

Uses of Interface sbrt.kernel.fba.comp.ExtremeCurrentComputer


|  |  |  |
| --- | --- | --- |
| **Overview** \| **Package** \| **Class** \| **Use** \| **Tree** \| **Deprecated** \| **Index** \| **Help** | |  |
| PREV   NEXT | **FRAMES**    **NO FRAMES**     **All Classes** |  |


---


## **Uses of Interface sbrt.kernel.fba.comp.ExtremeCurrentComputer**

| Packages that use ExtremeCurrentComputer | |
| --- | --- |
| **sbrt.kernel.fba.comp** | Provides classes and interfaces for performing Flux Balance Analysis computations. |
| **sbrt.kernel.fba.proc** | Provides classes and interfaces for executing Flux Balance Analysis processes. |
| **sbrt.shell.mng.fba** | Provides classes and interfaces for creating Flux Balance Analysis process managers. |

Uses of ExtremeCurrentComputer in sbrt.kernel.fba.comp

| Classes in sbrt.kernel.fba.comp that implement ExtremeCurrentComputer | |
| --- | --- |
| `class` | `SlpExCurrComputerV2`             This class is an implementation of an extreme current algorithm. |

Constructors in sbrt.kernel.fba.comp with parameters of type ExtremeCurrentComputer

`SlpCycleComputer(ExtremeCurrentComputer computer)`
  
          Constructs a new steady state cycle computer from the given extreme
current computer.

Uses of ExtremeCurrentComputer in sbrt.kernel.fba.proc

Constructors in sbrt.kernel.fba.proc with parameters of type ExtremeCurrentComputer

`ExtremeCurrentProcess(IrrevRxnFluxome fluxome,
ExtremeCurrentComputer computer)`
  
          Constructs a new extreme current experiment.

Uses of ExtremeCurrentComputer in sbrt.shell.mng.fba

| Methods in sbrt.shell.mng.fba that return ExtremeCurrentComputer | |
| --- | --- |
| `static ExtremeCurrentComputer` | `ExtremeCurrentManager.getComputer(ManagerInput<java.lang.String,java.lang.String> input, java.io.PrintWriter out)`             Returns an extreme current computer created from the provided input. |

---


|  |  |  |
| --- | --- | --- |
| **Overview** \| **Package** \| **Class** \| **Use** \| **Tree** \| **Deprecated** \| **Index** \| **Help** | |  |
| PREV   NEXT | **FRAMES**    **NO FRAMES**     **All Classes** |  |


---
